# Engineering the Surface of Ti_3_C_2_ MXene Nanosheets for High Stability and Multimodal Anticancer Therapy

**DOI:** 10.3390/pharmaceutics14020304

**Published:** 2022-01-27

**Authors:** Chiranjeevi Korupalli, Kai-Long You, Girum Getachew, Akash S. Rasal, Worku Batu Dirersa, Mochamad Zakki Fahmi, Jia-Yaw Chang

**Affiliations:** 1 Department of Chemical Engineering, National Taiwan University of Science and Technology, Taipei 10607, Taiwan; chiranjeevikorupalli@gmail.com (C.K.); edwin7954213@gmail.com (K.-L.Y.); getmeg88@gmail.com (G.G.); akashrasal079@gmail.com (A.S.R.); worku.batu13@gmail.com (W.B.D.); 2 Department of Chemistry, Universitas Airlangga, Surabaya 60115, Indonesia; m.zakki.fahmi@fst.unair.ac.id

**Keywords:** MXenes, Ti_3_C_2_, surface modification, cancer therapy, phototherapy, starvation therapy, cooperative therapy

## Abstract

The surface of Ti_3_C_2_ MXene nanosheets (TC NSs) was first modified with the antioxidants sodium ascorbate (SA) and dopamine (DA) (DSTC NS) to improve their stability in oxidative and hydration environments and thereby improve their bioapplications. This novel approach not only improved MXene stability by arresting oxidation but also increased the available functional groups for further functionalization with various biomolecules. The DSTC NSs were then sequentially conjugated with enzyme glucose oxidase (GOx) and photosensitizer Ce6 to render the obtained CGDSTC NSs with glucose starvation and photodynamic therapeutic properties and thus attain high efficiency in killing cancer cells through the cooperative effect. The as-synthesized CGDSTC NSs demonstrated tremendous photothermal effect with conversion efficiency of 45.1% and photodynamic (ROS generation) properties upon irradiation with 808 and 671 nm lasers. Furthermore, it was observed that the enzymatic activity of CGDSTC NSs increased upon laser irradiation due to enhanced solution temperature. During in vitro studies, the CGDSTC NSs exhibited cytocompatability to HePG2 and HeLa cells under nonstimulus conditions. However, they elicited more than 90% cell-killing efficiency in the presence of glucose and laser irradiation via the cooperative effect between starvation therapy and phototherapy. These results indicate that CGDSTC NSs could be used as potential therapeutic agents to eradicate cancers with no or few adverse effects. This surface modification approach is also simple and facile to adopt in MXene-based research.

## 1. Introduction

Two-dimensional (2D) materials have been attracting great attention from the scientific community in diverse research fields, including biomedical research, due to their many fascinating and unique properties [1,2]. Since the discovery of graphene in 2004 [3], a variety of 2D nanomaterials, such as transition metal dichalcogenides (e.g., TiS_2_, MoS_2_, and WS_2_) [4,5,6], black phosphorus nanosheets [7], layered double hydroxides [8], graphitic carbon nitride [9], 2D boron nitride [10], 2D metal–organic frameworks [11], transition metal oxides [12], etc., have been developed and explored as cancer therapeutic agents. Recently, MXenes, a new 2D material consisting of transition metal carbides, nitrides, or carbonitrides, have been drawing widespread fascination in the field of photothermal tumor treatment (PTT) as therapeutic, imaging, and drug delivery agents because of their high light-to-heat conversion efficiency, high atomic number and paramagnetic behavior, and high surface area [13,14,15]. However, MXene phototheraml agents (PTAs) are very unstable in aqueous/oxidative environments as their terminal functional groups (–F, –OH, or –O) are vulnerable to oxidation and tend to aggregate in physiological solutions, for instance, simulated body fluid and phosphate-buffered saline, ultimately leading to low PTT efficacy. These current challenges are impeding the application of MXene-based nanoplatforms in biomedicine.

To overcome the abovementioned pitfalls and improve the stability and bioapplication of MXenes, their surface has been engineered with soybean phospholipid [14,16], polyethylene glycol [17], or cellulose hydrogel [18]. Even though these approaches were effective, the prospect of new approaches to eliminate or restrict the oxidation of MXene nanosheets remains elusive. Besides stability, the MXene surface also lacks suitable functional groups for functionalization with various biologically active components that can endow multifunctional properties and thereby broaden their biomedical application potential. Multifunctional photothermal agents are necessary because PTT alone cannot completely remove tumors, and the residual cancer cells may result in recurrence of tumors [19]. Thus, it is necessary to engineer the surface of MXenes with new antioxidant molecules that can not only arrest oxidation but also endow functional groups on the surface for further modification with biologically active compounds. In view of this concern, in this study, we propose a novel approach to obtain MXene PTAs that are rich in functional groups and are highly stable through surface engineering with both dopamine (DA) and sodium ascorbate (SA) for PTT of cancer. DA and its derivatives are known to demonstrate strong adhesion to any surface through various chemical and physical interactions. They also have antioxidative properties due to the presence of catechol moieties and possess various functional groups, such as amines [20]. Therefore, DA is an ideal molecule to enhance surface functional properties and minimize oxidation effects. SA is another widely available antioxidant that is used in the food and pharmaceutical industries as preservative. It can inhibit surface oxidation and improve colloidal stability upon surface functionalization [21].

Thermal resistance of cancer cells caused by overexpressed heat shock proteins (HSP) in tumor tissues is the leading factor for reduced PTT efficacy [22]. To reverse cancer cell thermoresistance, HSP inhibitors, such as small interfering ribonucleic acid gene [23], 17-allylamino-17-demethoxygeldanamycin [24], gambogic acid [25], 2-phenylethynesulfonamide [26], etc., are incorporated into PTAs and delivered to cancer cells. However, high doses of these drugs may result in toxic side effects. Furthermore, they work by inhibiting the activity of existing HSPs rather than reducing the production of HSPs. It is known that high concentration of adenosine triphosphate (ATP) in tumors is responsible for overexpressed HSPs [27]. Thus, inhibition of ATP production can downregulate HSP generation and thereby enhance cancer cell sensitivity towards hyperthermia. As the glycolytic pathway of tumors causes generation of ATP, draining of glucose levels in the tumor is an effective approach to lower ATP levels in cancer cells. Glucose starvation therapy (GST) involves conversion of glucose to gluconic acid through glucose oxidase (GOx) or GOx mimic nanozymes. It can lower ATP levels by draining the tumor glucose levels and improve PTT efficacy to tumor cells [28,29]. As GOx demonstrates optimal catalytic activity at temperatures in the range of 43–60 °C, PTT can enhance the efficacy of GST [29,30]. Moreover, PTT is known to improve tumor oxygen levels due to increased blood flow, thus enhancing the efficacy of photodynamic therapy (PDT) [31]. Therefore, development of a multifunctional therapeutic agent with PTT, PDT, and GST properties is an ideal strategy to facilitate high tumor-specific therapeutic efficacy.

**Scheme 1:**
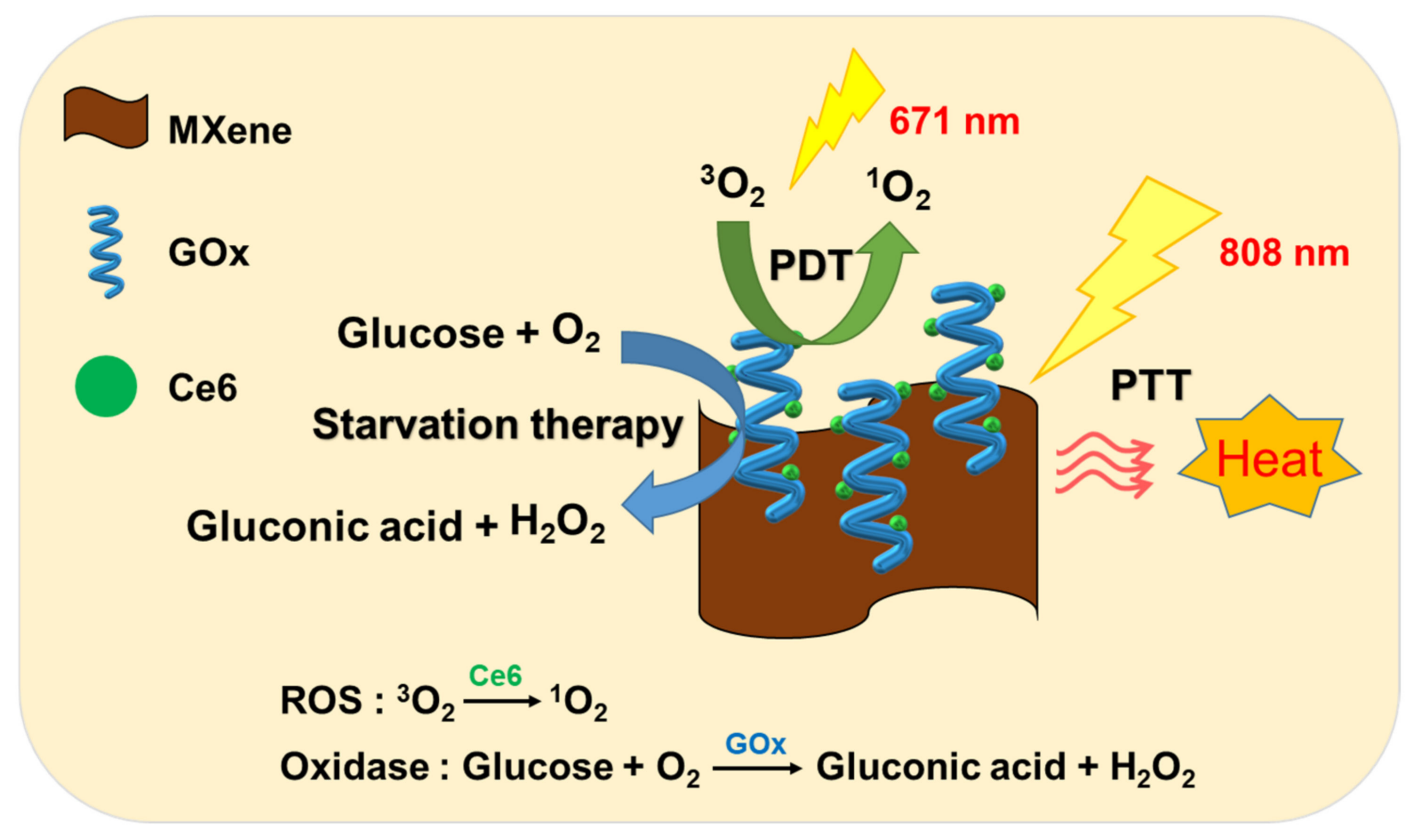
Pictorial representation for the working mechanism of CGDSTC NSs.

In this work, we used fluorescence (FL) imaging to develop MXene-based multifunctional theranostic nanosheets with photo and starvation therapeutic properties to attain high cancer therapeutic efficiency via the cooperative effect. We chose titanium carbide (Ti_3_C_2_) MXene as the PTA in this study. First, the stability of Ti_3_C_2_ MXene was enhanced through surface modification with the antioxidants SA and DA (DA/SA@Ti_3_C_2_ abbreviated as DSTC). SA and DA not only improved the stability of MXenes by arresting oxidation but also enabled functionalization with biological molecules. Then, GOx and Ce6 PS were sequentially attached onto the DSTC surface via simple carbodiimide coupling to empower it with GST and FL imaging and PDT properties, respectively (Ce6/GOx@SDTC abbreviated as CGDSTC). The working mechanism of CGDSTC NSs is shown in Scheme 1. Upon accumulation in glucose-rich tumor environments, the CGDSTC NSs could convert glucose into gluconic acid due to the presence of GOx and consequently sensitize the tumor cells to photothermal effects by constraining the tumor glycolytic pathway. Photo irradiation with 808 and 671 nm lasers led to destruction of tumor cells through Ti_3_C_2_- mediated PTT and Ce6-mediated PDT effects, respectively. Furthermore, laser irradiation with 808 nm laser augmented the efficiency of GST and PDT, with the higher temperature causing enhanced tumor oxygen levels and GOx catalytic activity. Moreover, the CGDSTC NSs allowed visualization of their localization into tumor cells due to FL imaging properties endowed by Ce6. Overall, high cancer-specific therapeutic efficiency was attained with our as-synthesized CGDSTC NSs via cooperative effect between starvation and phototherapy, which would effectually eliminate tumors in a more efficient and safer manner.

## 2. Materials and Methods

### 2.1. Chemicals and Materials

Titanium aluminum carbide (Ti_3_AlC_2_; 200 mesh) was obtained from 11 Technology (China). DA, GOx, Ce6, ethyl(dimethylaminopropyl)carbodiimide (EDC), WST-1, 9,10-dimethylanthracene (DMA), hydroxylamine (NH_3_O), triethylamine, D-(+)-Glucose, ethylenediaminetetraacetic acid (EDTA), minimum essential medium Eagle (MEM), Dulbecco’s modified Eagle’s medium with high glucose (DMEM), trypsin-EDTA solution (0.25%), L-glutamine solution, and antibiotic antimycotic solution were purchased from Sigma-Aldrich. SA was procured from Acros. Sulfo-NHS, trichloroacetic acid, and iron(III) chloride (FeCl_3_; 98%) were bought from Alfa Aesar. Fetal bovine serum (FBS) was obtained from Thermo Scientific HyClone (Logan, UT, USA). Titanium(IV) sulfate solution (Ti(SO_4_)_2_; 24%) was acquired from Kanto Chemical CO (Tokyo, Japan).

### 2.2. Instrumentation and Characterization

X-ray diffraction (XRD) spectra were obtained from D8 DISCOVER SSS multifunction spectrometer (Bruker). Scanning electron microscope (SEM) images were recorded with JSM-6500F (JEOL) and transmission electron microscope (TEM) images were recorded using Tecnai F20 G2 FEI-TEM (Philips). Fourier transform infrared (FT-IR) spectra were recorded in FTS-3500 (Bio-Rad CO. Ltd., Hercules, CA, USA). Zeta potentials were measured using Zetasizer 2000 (MALVERN). UV–Vis spectra were recorded using UV-1900I (SHIMADZU). Photoluminescence (PL) spectra were recorded in V-670 (JASCO CO. Ltd., Osaka, Japan).

### 2.3. Synthesis of Monolayer Ti_3_C_2_ MXene (TC)

Here, 100 mg of Ti_3_AlC_2_ and 48% of hydrofluoric acid (HF; 2 mL) were mixed and stirred in a Teflon container for 24 h. The obtained mixed solution was centrifuged at 6000 rpm for 10 min, and the precipitate was washed with DI water five times (until the pH of the solution became 7). The resultant multilayer TC (100 mg) was dispersed back in DI water (5 mL) and subjected to ultrasonication (amplitude = 60%, frequency 37 kHz) for 4 h to obtain black monolayer TC.

### 2.4. Synthesis of DSTC NSs

Multilayer TC NSs (200 mg) were dispersed in DI water (70 mL) and sonicated for 1 h. To this solution, SA (25 mM) was added and sonicated for 4 h under nitrogen atmosphere at 4 °C. The reaction solution was centrifuged (3500 rpm, 10 min) to remove large precipitates, and the supernatant was dialyzed (MW = 14,000) against DI water to remove free SA. The obtained STC NSs were mixed with DA powder (10:1) and stirred for 1 h under inert atmosphere. Subsequently, the reaction solution was dialyzed for 2 days against DI water to remove free DA. The obtained DSTC NSs were stored in a refrigerator.

### 2.5. Conjugation of GOx to DSTC NSs (GDSTC NSs)

To 1 mL of GOx (1 mg/mL) solution, 5 µL of EDC and sulfo-NHS (5 mg) were added and stirred for 1 h with a magnetic bar. Next, 5 mL of DSTC NSs was added and stirred for another 6 h to allow formation of amide linkage. Finally, the free GOx was removed through dialysis (100 KDa MW) of the reaction solution against DI water for 2 days. The obtained GDSTC NSs were stored at 4 °C.

### 2.6. Conjugation of Ce6 to GDSTC NSs (CGDSTC NSs)

Here, 1 mL of Ce6 (0.3 mg/mL DMSO) solution was mixed with EDC (5 µL) and NHS (5 mg) and stirred for 1 h to activate carboxyl groups. Then, 5 mL of GDSTC NS solution was added and stirred for another 6 h to allow formation of amide bond. The reaction mixture was dialyzed against DI water to remove DMSO and then centrifuged to remove free Ce6 by precipitation. The obtained CGDSTC NS solution was stored in refrigerator.

### 2.7. Determination of Photothermal Effect of CGDSTC NSs

The photothermal properties of as-synthesized CGDSTC NSs were investigated by placing aqueous solution (1 mL) into a quartz cuvette followed by irradiation with the 808 nm laser at a power density of 2 W/cm^2^ for 10 min. After every 30 s, the temperature changes were recorded by a thermocouple. CGDSTC NSs at different concentrations (0, 75, 100, 200, 300, 400, and 500 ppm), laser power densities (0.5, 1, 1.5, and 2 W/cm^2^), and final product of each synthetic procedure (STC, DSTC, GDSTC, and CGDSTC NSs) were used to examine the effect on photothermal performance. The photostability was investigated using five cycles of irradiation–cooling experiments. The photothermal conversion efficiency of the NSs was calculated by following a previously reported method [29,32].

### 2.8. Determination of Photodynamic Properties of CGDSTC NSs

The aqueous solution of GDSTC NSs or CGDSTC NSs (0.4 mL; 200 ppm) was mixed with DMA/DMSO solution (0.1 mL; 1.2 mM) and irradiated with the 671 nm laser (0.5 W/cm^2^) for 15 min with a 5 min interval. The fluorescence spectra were recorded after each irradiation (emission: 380–550 nm, excitation at 360 nm), and the changes in fluorescence intensity were compared to evaluate singlet oxygen generation.

### 2.9. Detection of H_2_O_2_ Concentration

The H_2_O_2_ concentration in the solution was measured by means of its reaction with Ti(SO_4_)_2_ reagent. First, 0.6 mL of 50 mM glucose, 1.5 mL of CGDSTC NSs (100 ppm), and 6.3 mL of PBS (pH 5.5) were mixed. Then, after every 10 min, 0.7 mL of the above solution was added to 1.4 mL of Ti(SO_4_)_2_ solution (1.33 mL of 24% Ti(SO_4_)_2_ and 8.33 mL of H_2_SO_4_ in 50 mL of DI water). Finally, the absorbance of the mixed solution at 405 nm was measured to determine the concentration of the H_2_O_2_ solution. To assess the effect of laser, the first solution was irradiated with 808 nm laser before mixing with the second solution.

### 2.10. Detection of Solution pH

To 80 µL of 50 mM glucose solution, 64 µL of 100 ppm CGDSTC NSs was added, and the pH of the obtained solution was measured using a pH meter (Hach sensIONTM). The solution was irradiated with laser before pH measurement to examine the effect of laser irradiation.

### 2.11. Detection of Dissolved O_2_

At room temperature, 0.8 mL of PBS buffer with a pH of 5.5 was added to 0.1 mL of 50 mM glucose and 0.2 mL of 100 ppm CGDSTC NSs, and the concentration of the dissolved O_2_ was measured with an oxygen meter (DO-5509).

### 2.12. Determination of Gluconic Acid Concentration

The gluconic acid concentration in the solution was determined through a colorimetric assay specific to gluconic acid. First, 15 μL of PBS with pH 5.5, 100 μL of 50 mM glucose, and 25 μL of CGDSTC NSs (100 ppm) were mixed. After 1 h reaction, solution 1 (5 mM EDTA + 0.15 mM Et_3_N solution; 125 μL) and solution 2 (3 M NH_2_OH; 12.5 μL) were added and reacted for 30 min. Then, solution 3 (1 M HCl + 0.1 M FeCl_3_ + 0.25 M CCl_3_COOH; 62.5 μL) was added and reacted for another 10 min. Finally, the absorbance (λ_505_) was measured.

### 2.13. Cytocompatibility of CGDSTC NSs

The WST-1 cell proliferation assay was employed to access cytocompatibility of the CGDSTC NSs. HeLa and HepG2 cells were used in this study. In the 96-well plate, the cells were seeded (5000 cells/well) and cultured for 24 h in an incubator (5% CO_2_) at 37 °C. Then, the cells were washed three times with PBS, and a medium containing CGDSTC NSs at different concentrations (0, 25, 50, 75, 100, and 200 ppm) was added before incubating for 24 or 48 h. At each time point, the medium was replaced with new medium containing WST-1 solution (10 μL/100 μL) and incubated for 4 h. The O.D. value at λ_450_ nm was measured and compared with control to evaluate cell viability.

### 2.14. Cellular Internalization and Intracellular ROS Detection

HeLa cells were cultured for 24 h in a 6-well plate (10,000 cells/well) encompassing a cover glass slip. Then, the culture medium was replaced with new medium containing predetermined concentration of CGDSTC NSs (0, 25, 50, and 75 ppm) and incubated for another 24 h. Subsequently, the medium was aspirated to remove the free material and washed with PBS. New medium containing 10 μM DCFH-DA was then added and incubated for 1.5 h. Free DCFH-DA was removed, new medium was added, and every well was irradiated with 671 nm (2 W/cm^2^) laser. Finally, the cells were imaged using confocal microscopy after staining the cell nucleus with DAPI. ImageJ software was used to quantify the fluorescence intensity.

### 2.15. In Vitro Therapeutic Efficacy of CGDSTC NSs

The HeLa cells were cocultured with CGDSTC NSs at different concentrations (0, 25, 50, 75, 100, and 200 ppm) in 96-well plates for 24 h under 5% CO_2_ atmosphere. Then, the cells were irradiated for 10 min with 808, 671, and both 808 and 671 nm lasers for PTT, PDT, and PTT + PDT effects, respectively. Next, 10 μL of WST-1 solution was added followed by incubation for 4 h, and the O.D. value of λ_450_ nm was measured. The cell viability was evaluated by comparing with control cells. For starvation effect, the cells were cultured in a medium containing 50 mM glucose.

## 3. Results and Discussion

### 3.1. Synthesis and Characterization of CGDSTC NSs

**Figure 1:**
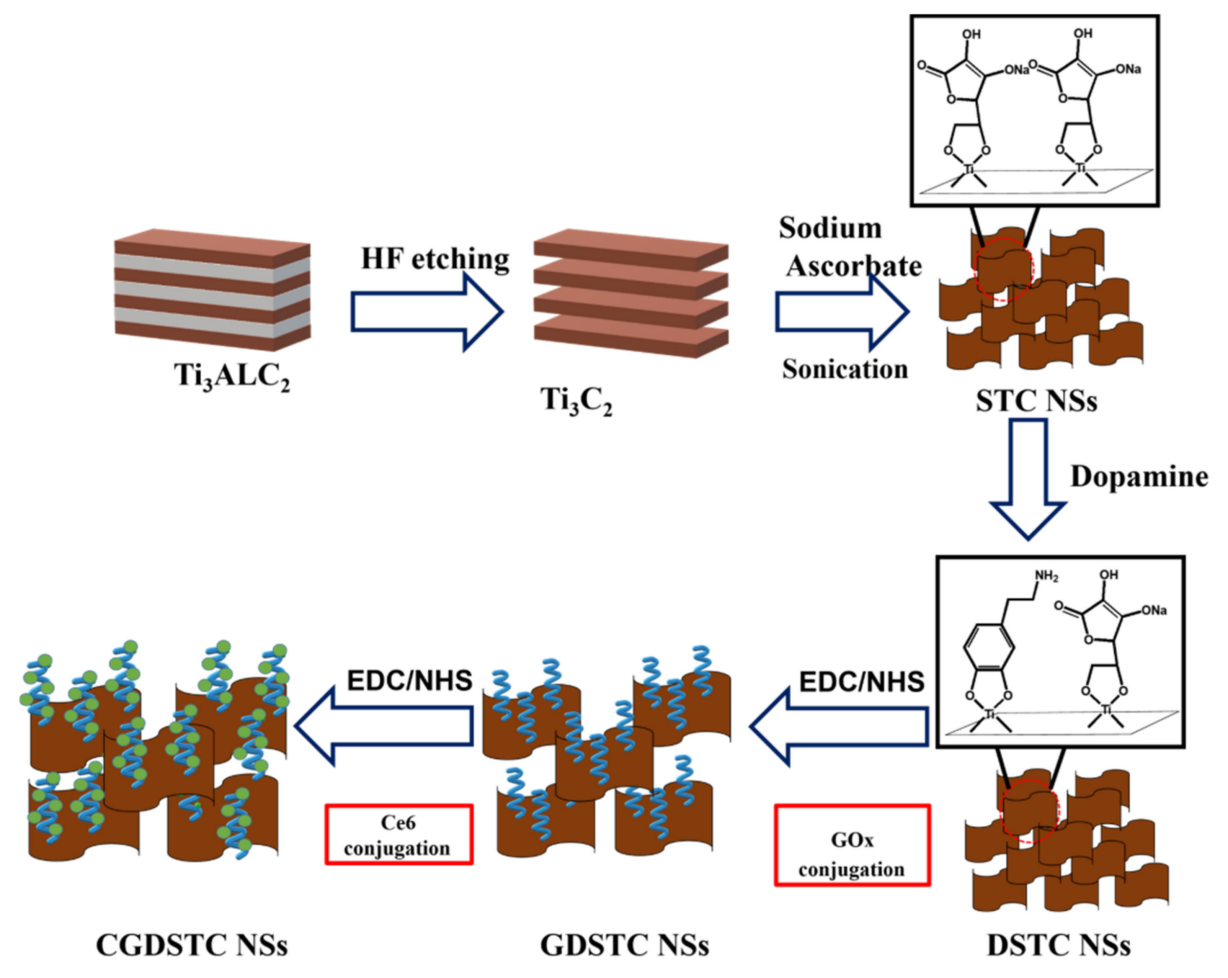
Schematic illustration for the fabrication process of CGDSTC NSs.

**Figure 2:**
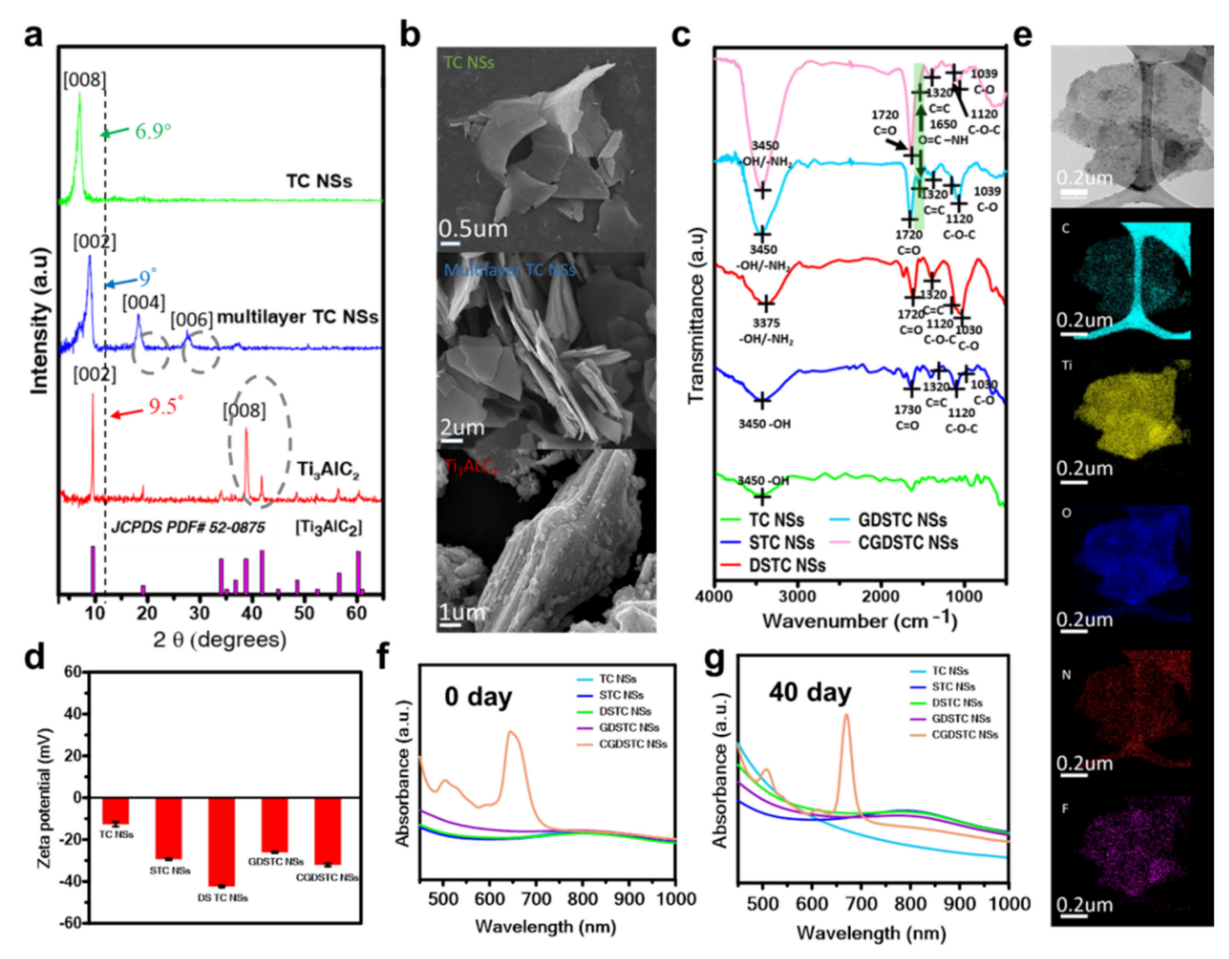
(**a**) XRD spectra and (**b**) SEM of Ti_3_AlC_2_, multilayer TC NSs, and TC NSs. (**c**) FT-IR spectra and (**d**) zeta potential measurements of TC NSs, STC NSs, DSTC NSs, GDSTC NSs, and CGDSTC NSs. (**e**) HRTEM and elemental mapping images (C, Ti, O, N, and F) of CGDSTC NSs. UV–Vis spectra of TC NSs, STC NSs, DSTC NSs, GDSTC NSs, and CGDSTC NSs (**f**) for 0 day storage and (**g**) after 40 days storage (stored at room temperature).

MXenes demonstrate much better light-to-heat conversion efficiencies than those of conventional photothermal agents, such as graphene and gold nanorods due to their large surface area [33]. Furthermore, in the MXene family, Ti3C2 MXene was the first to be discovered and is the most studied MXene. Hence, Ti3C2 MXene was chosen in this study. The schematic for the fabrication of CGDSTC NSs is shown in Figure 1. At the outset, the multilayered TC MXene NSs were fabricated by removing aluminum (Al) layers in Ti_3_AlC_2_ through selective etching with HF. The acquired multilayered TC NSs were dispersed into single-layered TC NSs by ultrasonication. The obtained TC NSs structures were analyzed using XRD spectroscopy. Upon etching with HF, the peak corresponding to (008) plane in Ti_3_AlC_2_ disappeared in the XRD spectra of multilayered TC NSs, indicating that the aluminum layer was removed by HF etching (Figure 2a) [16]. Moreover, the position of (002) plane shifted from 9.5° to 9.0° and the characteristic crystal planes (004) and (006) appeared, confirming the transformation of the tight structure to fan-shaped structure and the formation of multilayer TC NSs. The resultant TC NSs were ultrasonically processed to transform into single-layered TC NSs. In the XRD spectra of single-layered TC NSs structure, the characteristic peak of (002) plane shifted to 6.9° from 9.0° (Figure 2a). The SEM images (Figure 2b) recorded after every fabrication procedure further confirmed the structural changes of MXene NSs. Next, the DSTC NSs were fabricated through surface modification of multilayered TC NSs with SA and DA sequentially. In order to confirm the bonding of SA/DA onto TC NSs, zeta potential measurements and FT-IR analysis were performed. The TC NSs exhibited surface charge of −12.6 mV due to the presence of –OH functional groups on the surface (Figure 2d). After interaction with SA, the surface charge became more negative to −29.4 mV due to the added –OH and carboxyl functional groups from SA on the surface of NSs, signifying that SA molecules were adsorbed onto the surface of TC NSs. After interaction of STC NSs with DA, the surface charge of the obtained NSs became −42.4 mV, which was attributed to the adsorption of DA onto the surface as DA can generate catechol-rich surface after functionalization. In FT-IR spectra, the TC NSs showed a broad peak at 3450 cm^−1^ corresponding to –OH stretching (Figure 2c). However, in the FT-IR spectra of STC NSs, the peak at 3450 cm^−1^ became more intense after interaction with SA due to the high number of –OH functional groups. Furthermore, the characteristic peaks of SA at 1730, 1320, 1120, and 1030 cm^−1^ corresponding to C=O, C=C, COC, and CO bonds, respectively, appeared due to the adsorption of SA onto the surface of TC NSs. After interaction with DA, the intensity of peak at 3375 cm^−1^ became stronger than that of STC NSs, which might be due to the presence of –OH and –NH_2_ functional groups of DA. The zeta potential measurements and FT-IR analysis together confirmed that SA and DA were adsorbed onto the surface via noncovalent interactions and high number of functional groups, including –OH, –COO–, and –NH_2_, available on the surface for functionalization with biomolecules.

As the surface of DSTC NSs is rich in various functional groups, it is possible to functionalize them with different biomolecules to broaden their bioapplication. To this end, enzyme GOx and photosensitizer Ce6 were sequentially conjugated onto the DSTC NSs via carbodiimide coupling between the carboxyl groups and amine groups of DSTC NSs to achieve CGDSTC NSs with photodynamic and starvation properties. The successful conjugations were analyzed using zeta potential and FT-IR analysis. After reaction with GOx, the zeta potential of GDSTC NSs increased to −26.0 from −29.4 mV of DSTC NSs due to the presence of protonated amines in GOx, indicating the successful conjugation of GOx onto the DSTC NSs (Figure 2d). Likewise, after the reaction between GDSTC NSs and Ce6, the zeta potential of the resultant CGDSTC NSs decreased to −32.1 mV due to the carboxylic groups of Ce6, confirming the formation of CGDSTC NSs. In FT-IR, the spectra of GDSTC NSs exhibited a new peak at 1650 cm^−1^ corresponding to the newly formed amide bond (Figure 2c). The intensity of this peak became stronger upon conjugation with Ce6 due to the formation of more amide bonds. These outcomes proved the formation of CGDSTC NSs. The morphology of the as-synthesized CGDSTC NSs was observed under high-resolution TEM (HRTEM). As shown in Figure 2e, the CGDSTC NSs had sheet-like morphology, suggesting that the fabrication process did not affect the morphology of TC NSs. Furthermore, the EDS analysis indicated the existence of uniformly distrusted N elements along with Ti, C, O, and F elements in the structure (Figure S1, see Supplementary Materials). The N elements existed in the structure due to the conjugation with DA and GOx. The optical properties of the CGDSTC NSs were analyzed by measuring the UV–Vis spectra in the wavelength range of 450–1000 nm. As reported previously, the UV–Vis spectra of TC NSs exhibited strong absorbance in the NIR region of 750–1000 nm (Figure 2f). The UV–Vis spectra of surface-modified TC NSs, i.e., STC NSs, DSTC NSs, and GDSTC NSs, as well as CGDSTC NSs demonstrated NIR absorbance similar to TS NSs, indicating that the fabrication processes did not cause changes in the absorbance of TSs. However, the UV spectra of CGDSTC NSs showed an extra peak at around 670 nm because of the conjugation of Ce6.

It was anticipated that modification with SA and DA would enhance the stability of TC NSs. Thus, the storage stability of CGDSTC NSs was investigated by comparing the UV–Vis spectra of each composite obtained after 40 days storage at room temperature with those obtained for 0 day storage. The absorbance spectra of surface-modified TC NSs after 40 days storage were similar to those obtained for 0 day storage, indicating that surface-modified TCs are highly stable (Figure 2g). However, the unmodified TC NSs showed loss in NIR absorbance after 40 days storage compared to that for 0 day storage due to their instability (Figure 2g). Furthermore, the black-colored solution of unmodified TC NSs turned to white after 40 days storage due to oxidation, whereas the color of surface-modified TC NSs remained unchanged (Figure S2, see Supplementary Materials). Overall, these analytical results showed that surface modification of TC NSs with SA and DA not only increased the aqueous solubility and stability but also enabled conjugation with biomolecules.

### 3.2. Photothermal Properties of CGDSTC NSs

**Figure 3:**
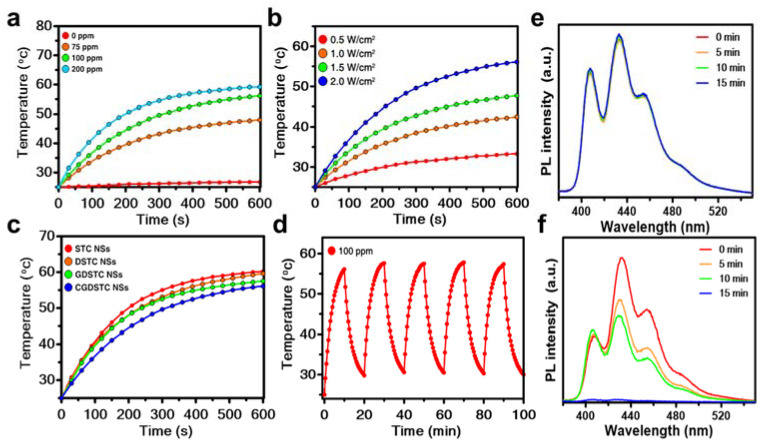
(**a**) Temperature elevation curves of CGDSTC NS solutions at different concentrations upon irradiation with 808 nm laser (2 W/cm^2^). Temperature elevation profiles of (**b**) CGDSTC NS solution (100 ppm) at different power densities of 808 nm laser and (**c**) STC NSs, DSTC NSs, GDSTC NSs, and CGDSTC NS solutions upon laser irradiation (808 nm, 2 W/cm^2^). (**d**) heating–cooling curves of CGDSTC NS solution (100 ppm) for five cycles of laser on/off (808 nm, 2 W/cm^2^). PL spectra of DMA with (**e**) GDSTC NSs and (**f**) CGDSTC NSs under 671 nm laser irradiation (0.5 W/cm^2^).

As the CGDSTC NSs demonstrated strong absorption properties in the NIR region (Figure 2f), their light-to-heat conversion abilities were investigated by illuminating them with 808 nm laser and then measuring changes in the solution temperatures. As shown in Figure 3a, pure water did not show notable changes in temperature even after 10 min laser irradiation (ΔT = 1.7 °C); however, the temperature of aqueous solutions containing CGDSTC NSs increased with increased irradiation time and almost reached plateau after 10 min of irradiation. Moreover, the final temperature of the solution increased as the concentration of CGDSTC NSs increased from 75 to 200 ppm. After 10 min of laser irradiation, the aqueous solutions containing 0, 75, 100, and 200 ppm of CGDSTC NSs exhibited final temperatures of 26.7, 47.5, 56.1, and 59.6 °C, respectively, from the initial temperature of 25 °C (Figure 3a). The irradiation studies also indicated that the increase in solution temperature was dependent on laser power density, and it was higher under high power density (Figure 3b). These results revealed that it was possible to control the solution temperature. Next, the effect of each fabrication step on the photothermal properties was investigated by irradiating each final composite compound. Each added molecule caused a very slight decrease in the final temperature of the solution compared to that of STC NSs (Figure 3c). For example, 100 ppm of STC NSs increased the solution temperature from 25 to 60.2 °C after 10 min irradiation, whereas the final CGDSTC NSs increased to 56.1 °C under similar conditions. This observation could be credited to the decrease in TC NSs content in 100 ppm of final composite compared to that in 100 ppm of STC NSs. By following the procedure reported elsewhere [32], the photothermal conversion of efficiency (η) for CGDSTC NSs was determined to be 45.1%. To qualify as a good photothermal agent, it should demonstrate excellent stabilities towards light activation. Thus, the CGDSTC NSs were made to undergo five cycles of laser on/off irradiation. As shown in Figure 3d, the change in solution temperature after five cycles of on/off irradiation was similar to that obtained upon initial irradiation, validating the photostability of the CGDSTC NSs. Overall, these analytical data confirm that the as-synthesized CGDSTC NSs are potential candidates for photothermal applications.

### 3.3. Photodynamic Properties of CGDSTC NSs

Ce6 is one of the well-known PSs, and its existence provides PDT properties to the corresponding composite materials, i.e., capability to generate reactive oxygen species (ROS) upon irradiation with light. Accordingly, ROS generation properties of the as-synthesized CGDSTC NSs under 671 nm were investigated by monitoring the fluorescence intensity of 9,10-dimethylanthracene (DMA). DMA is a fluorescent probe (λex = 375 nm and λem = 436) and loses its fluorescence after interaction with singlet oxygen (^1^O_2_) by forming 9,10-endoperoxide; thus, it has frequently been used as chemical trap for ^1^O_2_. As presented in Figure 3f, in the presence of CGDSTC NSs (100 ppm) and 671 nm laser, the fluorescence intensity of DMA gradually decreased with increased irradiation time. The fluorescence of DMA was completely quenched within 15 min of irradiation, whereas GDSTC NSs and laser did not cause notable changes to the fluorescence intensity of DMA (Figure 3e), thus validating that Ce6 is the key component for PDT properties of CGDSTC NSs.

### 3.4. Enzymatic Activities of CGDSTC NSs

**Figure 4:**
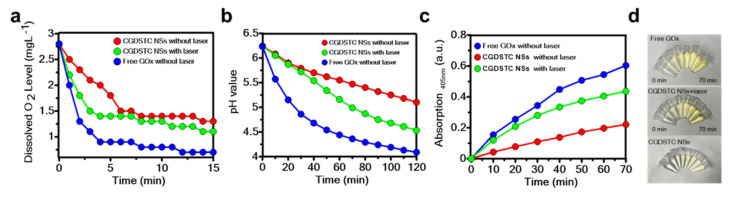
Time-dependent changes in (**a**) dissolved oxygen levels and (**b**) pH of glucose solution in the presence of free GOx, CGDSTC NSs, and CGDSTC NSs + laser. (**c**) Time-dependent changes in absorbance of fixed wavelength (λ = 405 nm) in the presence of free GOx, CGDSTC NSs, and CGDSTC NSs + laser (analysis of H_2_O_2_ production). (**d**) Photographs to show the time-dependent generation of H_2_O_2_ under free GOx, CGDSTC NSs, and CGDSTC NSs + laser.

The CGDSTC NSs are assumed to demonstrate GOx enzymatic properties due to the presence of GOx enzyme. Generally, in the presence of enzyme GOx, glucose converts to gluconic acid and H_2_O_2_ by consuming surrounding oxygen, thereby resulting in a significant drop in the solution pH and oxygen levels and an increase in solution H_2_O_2_ concentration due to the increase in enzyme–substrate interaction time. Accordingly, the glucose conversion properties of CGDSTC NSs were investigated by measuring the solution pH, concentration of H_2_O_2_, and dissolved O_2_ levels. Free GOx was used as positive control. As the mixing time between glucose and CGDSTC NSs increased, the pH and dissolved O_2_ levels of the resultant solution gradually decreased (Figure 4a,b). At the same time, the concentration of H_2_O_2_ in the solution gradually increased (Figure 4c).

These results showed a similar trend to that obtained in the presence of free GOx enzyme (Figure 4), confirming that the attached GOx conferred the CGDSTC NSs with enzymatic activities and that the fabrication approach did not cause significant changes to the enzyme GOx activity. The effect of laser irradiation on the enzymatic activities of CGDSTC NSs was further investigated. In the presence of laser irradiation, at any time point, the solution pH and O_2_ levels were lower and H_2_O_2_ levels were higher compared to those observed in the absence of laser. These results suggest that laser irradiation enhanced the enzymatic activity, which can be attributed to increased solution temperature due to the photothermal effect of CGDSTC NSs. The enzymatic activity is known to increase under high temperatures. Overall, the as-synthesized CGDSTC NSs are potential candidates to exhibit the cooperation effect between phototherapy and starvation therapy. Furthermore, the kinetic parameters and the catalytic activities were analyzed by performing steady-state kinetics study in the presence of glucose and CGDSTC NSs/free GOx/CGDSTC NCs + laser. The gluconic acid formation reaction in all the samples exhibited typical Michaelis–Menten curves within the appropriate range of glucose concentrations (Figure S3, see Supplementary Materials). The kinetic parameters of Michaelis–Menten constant (Km) and maximal velocity (Vmax) were obtained from the Lineweaver–Burk model and are shown in Table S1 (see Supplementary Materials). At the same concentration of CGDSTC NSs, the Km and Vmax values (2.07 mM and 1476.3 mM/s) were deduced to be higher in the presence of laser than those obtained without laser (1.7 mM and 1351.9 mM/s), confirming that enzymatic activity is faster in the presence of laser. Moreover, as the concentration of glucose increased, the color of the solution became more brown (Figure S4, see Supplementary Materials) due to the formation of more gluconic acid, which is consistent with increased catalytic activity.

### 3.5. In Vitro Cytocompatibility of CGDSTC NSs

**Figure 5:**
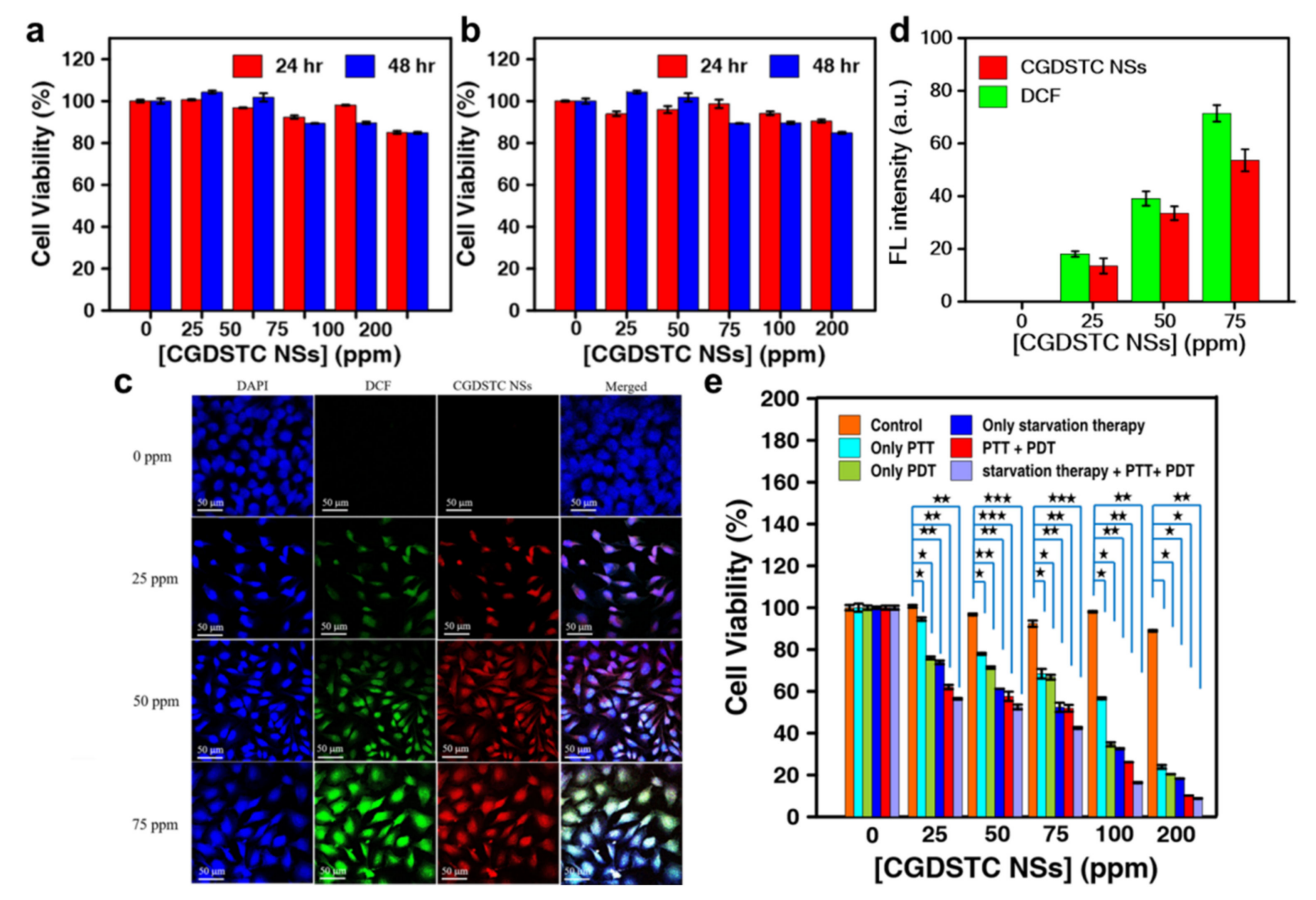
Cell viability of (**a**) HeLa and (**b**) HepG2 cells after 24 and 48 h treatment with different concentrations of CGDSTC NSs. (**c**) Confocal microscopy images of HeLa cells treated with different concentrations of CGDSTC NSs (for 24 h) and 671 nm laser (0/5 W/cm^2^, 10 min) and (**d**) corresponding quantified fluorescence intensities. (**e**) Cell viability of HeLa cells at different concentrations of CGDSTC NSs under different treatment conditions, namely PTT alone, PDT alone, starvation alone, PTT + PDT, and PTT + PDT + starvation (^★^ *p* < 0.05, ^★★^ *p* < 0.01, ^★★★^ *p* < 0.001).

It is always necessary to evaluate the possible nonspecific toxicity of therapeutic agents before considering them for treatment in order to provide safer therapy to the convalescent. Therefore, the cytocompatibility of the as-synthesized CGDSTC NSs was examined by coculturing them with cervical cancer cells (HeLa cells) and human liver cancer cells (HepG2 cells) for 48 h. WST-1 assay was employed for quantitative evaluation of cytotoxicity. As depicted in Figure 5a,b, the viabilities of cells treated with CGDSTC NSs did not vary significantly from those of the untreated cells within the given incubation periods (0–48 h) and concentrations (0–200 ppm), indicating that NSs are biologically safe under nonstimulus conditions and can be exploited for site-specific therapeutic activity with no or few adverse effects.

### 3.6. Cellular Internalization of CGDSTC NSs

Ce6 is also a red-emitting fluorescent probe [34]. Thus, it is anticipated that Ce6 containing CGDSTC NSs could also be used as fluorescence imaging agents to monitor their homing into cells. Hence, the cellular internalization of CGDSTC NSs was investigated by imaging NS-treated HeLa cells under confocal microscopy. DAPI was used to stain the nucleus of the cells. After 24 h incubation, the cells treated with NSs exhibited strong red fluorescence under confocal microscopy, whereas untreated cells did not exhibit any red fluorescence (Figure 5c). Furthermore, the red fluorescence overlaid the blue fluorescence, indicating that CGDSTC NSs were effectively internalized into the cell nucleus. As the concentration of incubated NSs increased, the intensity of red fluorescence increased because more NSs were internalized into cells (Figure 5d). Overall, these outcomes indicate that CGDSTC NSs can also be employed for fluorescence imaging guided therapy.

### 3.7. Intracellular ROS Detection

It was validated that CGDSTC NSs could generate ROS upon laser irradiation (Figure 3f) with 671 nm laser. Thus, the intracellular ROS in the cells after treatment with CGDSTC NSs and laser was detected using a DCFH–DA assay kit. Cells were treated with the as-synthesized NSs and DCFH–DA and then imaged using confocal microscopy after laser irradiation. In this study, untreated cells were used as the control, and DAPI was used for the staining of cell nucleus. As shown in Figure 5c, the control cells did not exhibit green fluorescence of DCF, indicating that ROS was not generated. However, the cells treated with CGDSTC NSs demonstrated green fluorescence due to conversion of DCFH-DA to DCF by ROS, suggesting that the internalized NSs generated ROS in the cells after laser irradiation. The intensity of green fluorescence increased as the concentration of incubated NSs increased (Figure 5d), indicating that more ROS was generated due to internalization of more NSs into the cells. These results validate that the as-synthesized NSs are effective in inducing ROS-mediated cancer cell death.

### 3.8. Cooperative Effect of NSs on Cancer Cell Death

In the above experiments, it was proven that CGDSTC NSs are compatible to cells under nonstimulus conditions and that they are able to aggravate cell-killing features, such as hyperthermia, reduced glucose concentrations, and ROS. Hence, the cell-killing efficiency of the as-synthesized NSs under the influence of various stimuli was studied either individually or in combination.

To verify the photothermal and photodynamic effect of NSs on cell death, HeLa cells were irradiated with 808 (2 W/cm^2^) and 671 (0.5 W/cm^2^) nm lasers, respectively, for 10 min after incubation with different concentrations of NSs for 24 h. For the combination effect of PTT and PDT, both lasers were applied on the cancer cells at the same time. As depicted in Figure 5e, laser irradiation alone did not induce any cell death. Nevertheless, the cell death was concentration-dependent in the presence of both CGDSTC NSs and laser, which could be attributed to the concentration-dependent photothermal and photodynamic properties of NSs. As the concentration of NSs increased, the percentage of viable cells in all treatments decreased, and it was significantly less in the PTT and PDT combination compared to that in PTT or PDT alone. For 200 ppm of CGDSTC NSs, PTT alone and PDT alone induced 76% and 79% cell death, respectively, whereas the combination of PTT and PDT induced 90% cell death.

Glucose is the essential nutrient for cancer cell growth [35]. Thus, it is possible to induce cell death by glucose starvation in cells [36]. As CGDSTC NSs are able to decompose glucose and modulate oxidative stress in microenvironments, the ability of NSs to induce cell toxicity through glucose starvation was verified by varying its concentration in the culture medium. In the presence of glucose alone, the cell viability was comparable to that of the control, indicating that glucose did not cause cell toxicity (Figure 5e). However, in presence of glucose (50 µg/ML) and CGDSTC NSs, the cell viability significantly dropped compared to that of the control. This decrease was greater at higher concentration of NSs because of high catalytic activities, suggesting that glucose consumption of NSs caused cell death by inducing glucose starvation. At 200 ppm of NSs and 50 µg/mL of glucose, the cell viability was around 20%.

As phototherapy and glucose metabolism can lessen each other’s downsides, i.e., catalytic activity, therapeutic sensitivity, etc., and lead to amplified cancer cell damage through win–win cooperation [30], the synergistic effect of multiple therapeutic modalities of NSs on cell viability was further investigated. For this, HeLa cells were incubated with glucose (50 μg/mL) and various concentrations of NSs and irradiated with both 808 and 671 nm lasers for 10 min. At all the tested concentrations of NSs, the combined glucose starvation therapy and phototherapy exhibited superior cell death than that obtained with PTT, PDT, PTT + PDT, or starvation therapy (Figure 5e). Overall, these results suggest that CGDSTC NSs have the potential to become promising therapeutic agents in cancer treatment.

## 4. Conclusions

In this work, the surface of Ti_3_C_2_ MXene (TC) NSs was modified with the antioxidants sodium ascorbate (SA) and dopamine (DA) to enhance their stability in oxidative and hydration environments. This approach not only improved the stability but also enabled conjugation of glucose oxidase (GOx) and photosensitizer (Ce6) onto the surface of TC NSs by increasing the available functional groups. The as-synthesized CGDSTC NSs demonstrated excellent photon-to-heat conversion, ROS generation, and glucose decomposition properties thanks to the presence of TC NSs, Ce6, and GOx, respectively. The CGDSTC NSs were effectively internalized into the cells, and the internalized NSs increased the intracellular ROS concentrations upon laser irradiation. Furthermore, the as-synthesized CGDSTC NSs were found to be cytocompatible under nonstimulus conditions but exhibited higher cancer cell toxicity in the presence of stimulus factors such as laser and glucose. The cancer cell toxicity was significantly higher with the combination of glucose starvation and phototherapy than that obtained with glucose starvation or phototherapy alone. From the results of this study, it is hypothesized that CGDSTC NSs could be potential therapeutic agents in cancer treatment. Moreover, the surface modification approach could be adopted in future MXene research as it is facile and easy.

## Data Availability

The data presented in this study are available upon request from the corresponding author.

## Supplementary Materials

The following are available online at https://www.mdpi.com/article/10.3390/pharmaceutics14020304/s1, Figure S1: EDS spectrum of CGDSTC NSs, Figure S2: Photographs of solutions after 0 day and 40 days storage, Figure S3: (a) Michaelis−Menten curves and (b) Lineweaver−Burk models for the generation of gluconic acid in the presence of free GOx (Blue dot), CGDSTC NSs+laser (Green dot), and CGDSTC NSs (Red dot), Figure S4: Photographs to show the generation of gluconic acid at different conditions, Table S1: The Vmax and Km values at different conditions.
