# Supplementary material for: Engineering the Surface of Ti3C2 MXene Nanosheets for High Stability and Multimodal Anticancer Therapy
Source: Pharmaceutics. 2022 Jan 27;14(2):304. doi: 10.3390/pharmaceutics14020304 (PMC8879045; doi:10.3390/pharmaceutics14020304)
Supplement: Supplementary file 1 [file pharmaceutics-14-00304-s001.zip › pharmaceutics-1553789-supplementary FINAL.pdf]

pharmaceuticals

MDPI

Supplementary Materials: Engineering the Surface of Ti_3C_2 MXene Nanosheets for High Stability and Multimodal Anti-cancer Therapy

Chiranjeevi Korupalli, Kai-Long You, Girum Getachew, Akash S. Rasal, Worku Batu Diresa, Mochamad Zakki Fahmi and Jia-Yaw Chang

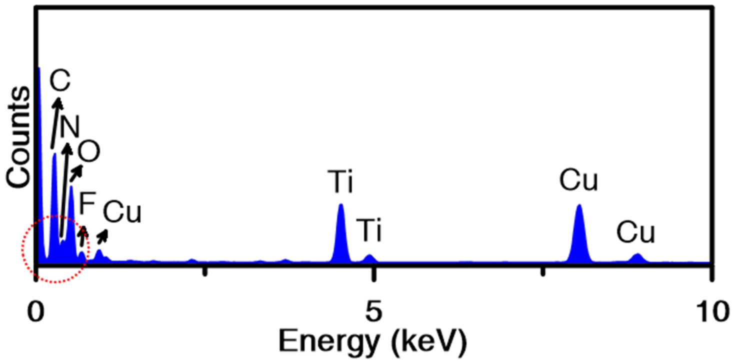

Figure S1. EDS spectrum of CGDSTC NSs.

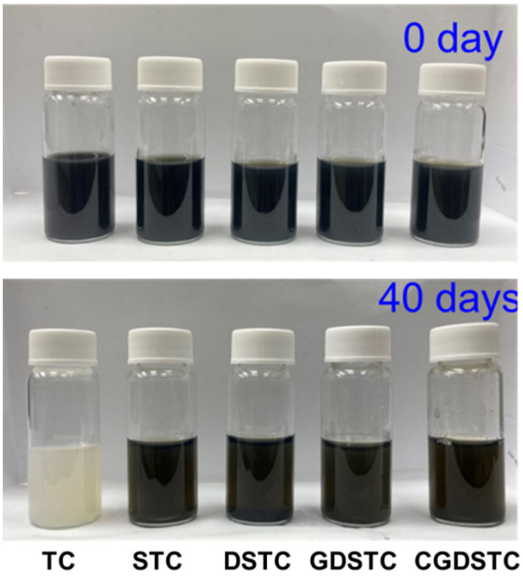

Figure S2. Photographs of solutions after 0 day and 40 days storage.

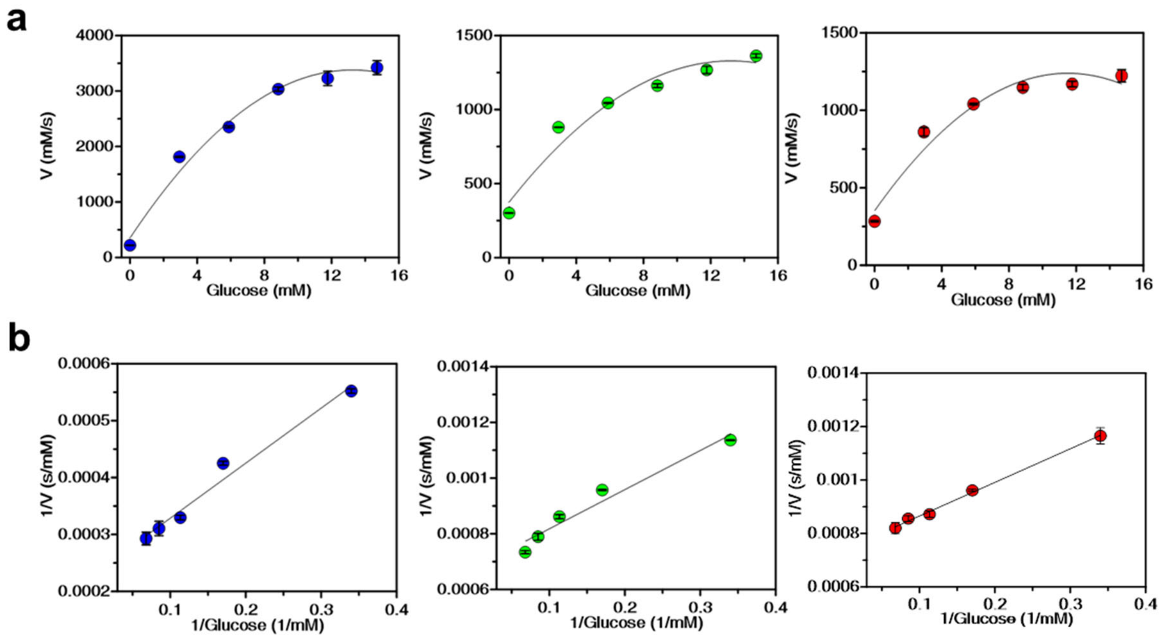

Figure S3. (a) Michaelis−Menten curves and (b) Lineweaver−Burk models for the generation of gluconic acid in the presence of free GOx (Blue dot), CGDSTC NSs+laser (Green dot), and CGDSTC NSs (Red dot).

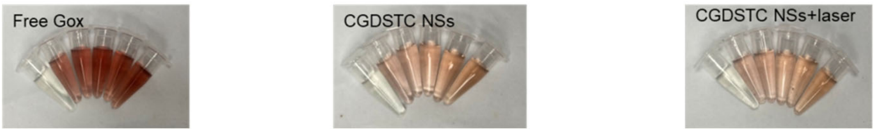

Figure S4. Photographs to show the generation of gluconic acid at different conditions.

Table S1. The Vmax and Km values at different conditions.

	V_{max} (mM/s)	K_m (mM)
Free Gox without laser	4311.2	4.17
Ce6-Gox-MXene with laser	1476.3	2.07
Ce6-Gox-MXene without laser	1351.9	1.7
